# 2018 consensus statement by the Spanish Society of Pathology and the Spanish Society of Medical Oncology on the diagnosis and treatment of cancer of unknown primary

**DOI:** 10.1007/s12094-018-1899-z

**Published:** 2018-05-28

**Authors:** F. Losa, L. Iglesias, M. Pané, J. Sanz, B. Nieto, V. Fusté, L. de la Cruz-Merino, Á. Concha, C. Balañá, X. Matías-Guiu

**Affiliations:** 1 Medical Oncology Department, Institut Català d’Oncologia ICO-Hospitalet/Hospital de Sant Joan Despí, Moisès Broggi, Sant Joan Despí, Carrer de Jacint Verdaguer, 90, 08970 Sant Joan Despí, Barcelona España; 2 Medical Oncology Department, Hospital Universitario Doce de Octubre, Madrid, Spain; 3 Pathology Department, Hospital Universitari de Bellvitge, L’Hospitalet de Llobregat, Barcelona, Spain; 4 Pathology Department, Hospital Clínico San Carlos, Madrid, Spain; 5 Medical Oncology Department, Hospital Universitario de León, León, Spain; 6 Pathology Department, Hospital de la Santa Creu i Sant Pau, Barcelona, Spain; 7 Medical Oncology Department, Hospital Universitario Virgen Macarena, Seville, Spain; 8 Pathology Department, Hospital Universitario A Coruña, La Coruña, Spain; 9 Medical Oncology Department, Institut Català d’Oncologia, Badalona, Barcelona Spain; 10 Pathology Department, Hospital Universitario Arnau de Vilanova de Lleida y Hospital Universitari de Bellvitge, IRBLLEIDA, IDIBELL, CIBERONC, L’Hospitalet de Llobregat, Barcelona, Spain

**Keywords:** Cancer of unknown primary, Diagnosis, Immunohistochemistry, Biopsy, Prognosis, Chemotherapy, Molecular pathology, Histopathology

## Abstract

Cancer of unknown primary (CUP) is defined as a heterogeneous group of tumours that present with metastasis, and in which attempts to identify the original site have failed. They differ from other primary tumours in their biological features and how they spread, which means that they can be considered a separate entity. There are several hypotheses regarding their origin, but the most plausible explanation for their aggressiveness and chemoresistance seems to involve chromosomal instability. Depending on the type of study done, CUP can account for 2–9% of all cancer patients, mostly 60–75 years old. This article reviews the main clinical, pathological, and molecular studies conducted to analyse and determine the origin of CUP. The main strategies for patient management and treatment, by both clinicians and pathologists, are also addressed.

## Introduction

Cancer of unknown primary (CUP) is defined as a heterogeneous group of tumours that present initially with metastasis, and in which a properly standardised diagnostic work-up cannot identify the original site of the malignancy [1, 2]. In the last few years, various consensus statements and international clinical guidelines have tried to define more clearly what diagnostic work-up should be performed before a case is regarded as CUP. The US National Cancer Institute (NCI), the National Comprehensive Cancer Network (NCCN), and the European Society of Medical Oncology (ESMO) have published minimum recommendations on this subject, with a reminder that any cancer presenting with metastasis must undergo the initial testing before being regarded as CUP [3, 4].

CUP can be considered a separate entity, because its biological properties set it apart from other known primary tumours. In addition, it generally has atypical patterns of spread and clinical behaviour, which are inconsistent with the supposed site of origin [5]. There are two hypotheses regarding CUP biology: the first suggests that the neoplasm might arise from a stem cell, without first producing a premalignant lesion or a primary tumour; the second maintains that it represents rapid progression of metastasis from a very early primary tumour [6].

Chromosomal instability was recently suggested as a plausible explanation, or even a prognostic factor for more aggressive presentation and chemoresistance of CUP [7]. It has been shown that, generally, CUP is not associated with specific mutations in oncogenes or suppressor genes. Instead, it is characterised by angiogenesis activation (50–89%), oncogene over-expression (10–30%), a greater presence of hypoxia-related proteins and epithelial–mesenchymal transition markers (16–25%), and the activation of intracellular signals such as AKT or MAPK (20–35%) [8].

Depending on the definition used and how exhaustive the diagnostic procedures are, CUP can account for 2% to 9% of all cancer patients [1]. Accordingly, the incidence of CUP may change as new diagnostic technologies are implemented. In purely numerical terms, CUP is currently the eighth most frequent cancer diagnosis [1]. Its incidence is highest in patients aged 60–75 years [9, 10]. The most common underlying occult primary tumours are basically of lung and biliopancreatic origin, as observed in the pooled analysis of over 800 patients in 12 autopsy series [11].

## Basic clinical work-up

The initial clinical assessment of patients diagnosed with CUP should not be exhaustive. Instead, it should aim to determine the extent of the disease, and to identify tumour subtypes in which a specific therapy may have a positive impact on patient progress and prognosis. The basic clinical investigations that should be done include history-taking and physical examination, basic laboratory tests, and computed tomography (CT) of the chest, abdomen, and pelvis.

A full medical history and physical examination should include genitourinary and rectal examination, as well as examining the breasts and pelvic region in women, and the prostate in men. Special attention should be paid to the previous diseases, biopsies or lesions removed, spontaneously regressing lesions, previous imaging tests, and family cancer history. The laboratory tests recommended are a full blood count with differential white cell count, measurement of electrolytes, creatinine and calcium, liver function tests, and basic urinalysis [4, 12].

Serum tumour markers should not be tested routinely. Elevations in these markers are not highly sensitive or specific, and testing for them during CUP diagnosis has not been shown to be cost-effective. In men, however, tests for prostate-specific antigen (PSA), serum chorionic gonadotropin (β-hCG), and alpha-fetoprotein (AFP) are recommended, to rule out treatable extragonadal germ cell tumours or prostate cancer eligible for endocrine therapy. In the case of adenocarcinomas with peritoneal involvement in women, a CA 125 test should be done [2, 13].

As far as the initial radiological tests are concerned, patients should have a contrast-enhanced CT scan of the chest, abdomen, and pelvis. If there are cervical lymphadenopathies, this should be accompanied by a CT scan of the neck. Positron emission tomography (PET) can be useful in certain clinical presentations, for both diagnosis and staging [14]. It has 87% sensitivity and 71% specificity [15]. One of the limitations of PET is its moderate precision regarding anatomical sites or functional abnormalities, because of low tracer uptake by some tumour tissues. In these cases, it is more helpful to combine PET with CT. A meta-analysis and systematic review of combined PET/CT in patients diagnosed with CUP found that primary tumours were detected in 37% of patients, with 84% sensitivity and identical 84% specificity [16]. This primary tumour detection rate is higher in the case of specific metastatic scenarios, such as cervical lymphadenopathies, and sensitivity is higher too [17]. However, in the only prospective study done, PET/CT proved no superior to CT [18]. PET is, therefore, not recommended as the initial test. Its use should be confined to specific clinical presentations (single metastases and cervical lymphadenopathies), and when local or regional therapy is being considered.

In women, it is helpful to perform mammography, and/or magnetic resonance imaging of the breasts if the mammography result is equivocal [13]. On the other hand, endoscopy should not be routinely employed, because it rarely detects the primary tumour in asymptomatic patients, and false positives can cause confusion [19]. The choice of other diagnostic procedures should be based on interpretation of the histological sample obtained by biopsy.

## Pathological diagnosis

### Optimising the sample

Obtaining a sample of enough tissue is essential for diagnosing and investigating CUP, both for tumour typing and for conducting additional molecular studies. The sample should be obtained using a procedure that provides as much tissue as possible but is not highly invasive for the patient [20]. Although the minimum number of cells needed for histological and molecular investigations is not well established, samples containing at least 400 cells are recommended [21]. Some studies show that cytology specimens can be diagnostically useful [22, 23]. To enable all the necessary tests to be done, however, solid tissue samples obtained by core-needle biopsy (CNB), incisional biopsy, or surgical resection are preferable.

On the other hand, it is essential to optimise the material obtained. The available material should be divided between multiple paraffin blocks, to conserve as much tissue as possible. Diagnostic immunohistochemical tests should be confined to reasonable panels, according to the available clinical and radiological evidence, to prevent unnecessary staining. In cases likely to require molecular techniques, it is advisable to prepare a few blank slides when serial sections are cut for routine immunostaining.

### Basic histopathology study and classification by morphological/histopathological subtype

After the initial clinical assessment, the sample is examined using histopathological techniques, which may require a basic immunohistochemical (IHC) panel. Preliminary classification then becomes possible [2, 24–26]:

#### Initial classification

The most common initial classifications are:

- Carcinoma/neuroendocrine tumour:
  - adenocarcinoma (60%);
  - poorly differentiated carcinoma, including poorly differentiated adenocarcinoma (20%);
  - squamous cell (epidermoid) and/or transitional cell carcinoma (5–10%);
  - neuroendocrine tumour (5%);
  - undifferentiated carcinoma;
- lymphoma;
- extragonadal germ cell tumour;
- melanoma;
- sarcoma.

In this first classification, it should be remembered that CUP may be due to tumours of atypical site, morphology, and immunophenotype. The most common metastases at that site have to be ruled out. Tumours that have effective specific therapies must also be appropriately excluded, such as germ cell tumours, neuroendocrine tumours, lymphoma, and hormone-dependent tumours (prostate in men and breast in women). Moreover, IHC markers may have limited specificity and sensitivity in this first classification.

#### General histopathological patterns

The general histopathological patterns are as follows:

- Epithelial/epithelioid: carcinoma, “epithelioid” sarcoma, and melanoma;
- Spindle cell: sarcoma, sarcomatoid carcinoma, and melanoma;
- Small cell: lymphoma, sarcoma, neuroendocrine tumour, primitive neuroectodermal tumour (PNET), and melanoma;
- Pleomorphic: all.

#### Second classification: most likely primary tumour

In poorly differentiated carcinomas and adenocarcinomas, immunohistochemistry is essential. In squamous cell carcinomas, morphology and immunohistochemistry are relatively non-specific, although there are subtypes associated with human papillomavirus that have greater specificity.

In some cases, relevant differences may exist between primary tumours and their metastases. For example, compared with primary tumours, melanoma metastases show greater cytokeratin (CK) AE1/AE3 staining (22%) and less HMB45 staining (65%). The marker S100 may be expressed in adenocarcinomas of the lung, breast, endometrium, and kidney (up to 80%), and other sites to a lesser extent [27].

Certain epithelioid or spindle cell sarcomas can be confused with carcinomas or melanomas. Many sarcomas have specific molecular alterations that can be demonstrated by immunohistochemistry or fluorescence in situ hybridisation (FISH) [28]. Molecular diagnostics are particularly recommended for sarcomas of atypical morphology or site.

#### Prognostic classification of CUP

The prognosis is favourable in 15–20% of CUP cases [24, 25]. These tumours are more chemosensitive and overall survival is better. This category includes poorly differentiated midline carcinomas, peritoneal papillary adenocarcinomas in women, metastatic adenocarcinoma involving the axillary lymph nodes only, metastatic squamous cell carcinoma in the cervical lymph nodes, single-node metastases, poorly differentiated neuroendocrine carcinoma, resectable tumours, and germ cell tumours. In contrast, other cases of CUP have a poor prognosis. These include adenocarcinomas with liver metastases, non-papillary malignant ascites, and multiple brain, bone, or lung metastases.

### Specific histopathology study

#### Advisable minimum IHC panel

**Fig. 1:**
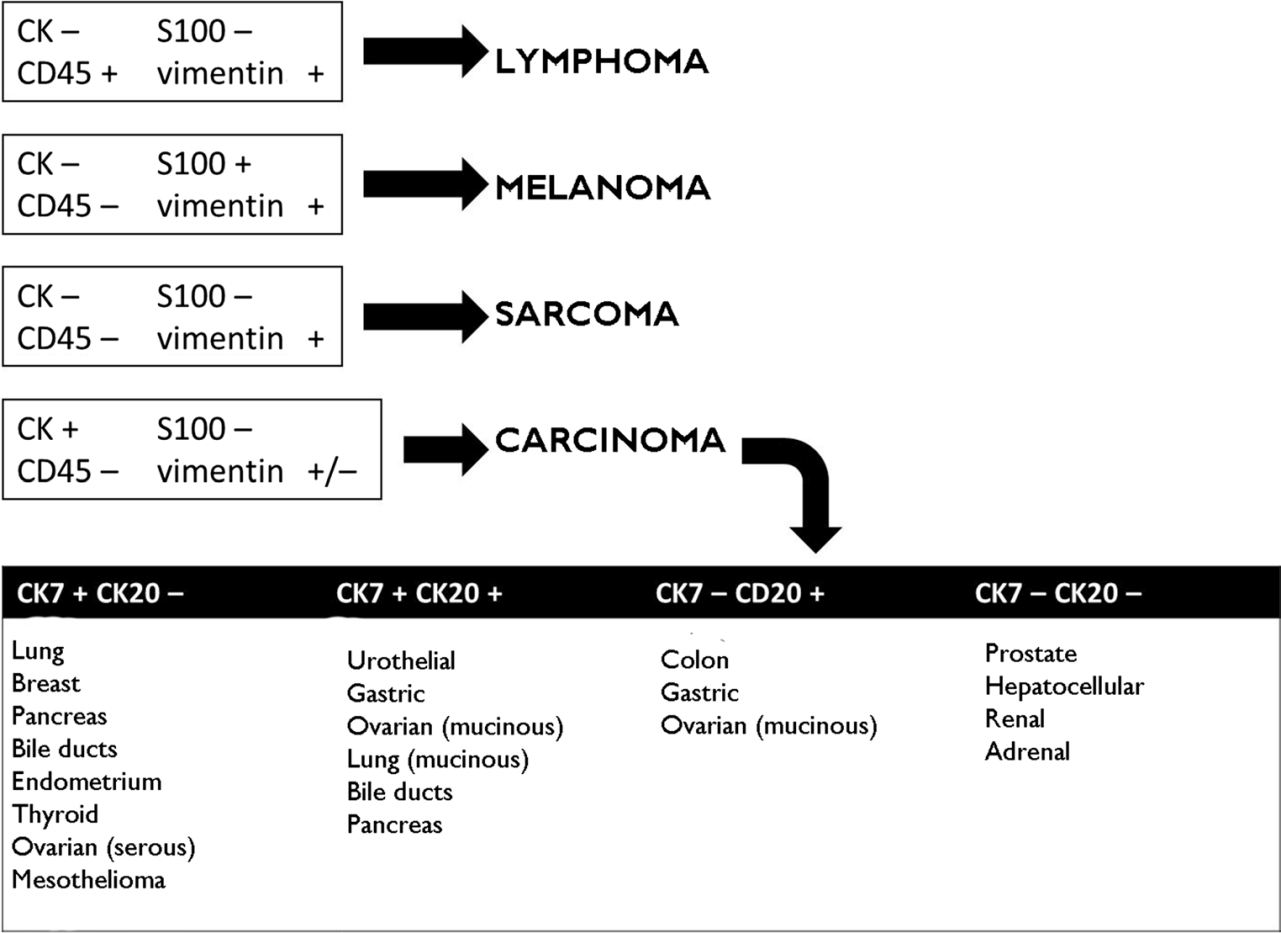
Advisable minimum IHC panel. *IHC* immunohistochemistry

The initial IHC panel should be based on the patient’s clinical details (age, sex, disease history, site, etc.) and morphological examination of the mass. For a general approach to CUP, the basic IHC panel should mainly consist of epithelial cell markers (CK AE1/AE3, CK Cam 5.2, EMA), S100, vimentin, and leucocyte common antigen (LCA or CD45) (Fig. 1). If epithelial origin of the lesion is confirmed (CK+ , S100−, vimentin ± and LCA−), a combination of CK7 and CK20 will give a first indication of the organ in which the tumour is most likely to have originated [29].

In general, although carcinomas express CK and mesenchymal tumours express vimentin, the exceptions to that rule must be remembered. For example, poorly differentiated carcinomas can show an unpredictable CK expression pattern, or CK expression may be lost completely (as in cases involving the kidney or adrenal gland), whereas some carcinomas co-express vimentin and CK (typically those found in the endometrium, thyroid, and kidney, among others). On the other hand, some types of sarcoma, such as epithelioid or synovial sarcomas, leiomyosarcomas or malignant peripheral nerve sheath tumours, can co-express vimentin and CK. This also occurs in polyphenotypic tumours/blastomas, such as Ewing sarcoma/PNET, desmoplastic small round cell tumour, or medulloblastoma.

Staining positive for CD45 and negative for CK suggests a lesion of haematological origin, although some haematolymphoid tumours (granulocytic sarcoma, myelomas, Hodgkin’s lymphoma, or anaplastic lymphomas) may have very little or no CD45 expression and test positive for S100 or EMA. S100 is highly specific for lesions of melanocytic or neural origin and may be expressed in a nuclear and cytoplasmic pattern. Vimentin is an intermediate filament characteristic of mesenchymal cells. It is found in almost all sarcomas, melanomas, and a good proportion of lymphomas. It tends to play a supportive role together with other markers.

The detection of tumours with neuroendocrine differentiation and tumours of germ cell origin deserves special mention. For neuroendocrine tumours, the panel should include synaptophysin, chromogranin A, and CD56. For germ cell tumours, a specific panel should be considered, such as PLAP, alpha-fetoprotein, or OCT 3/4. When the tumour’s microscopic features show it to be clearly epithelial, some of the markers in this profile can be omitted. In that case, the initial panel can be restricted to CK7 and CK20, and testing completed with the extended panel.

#### Optional extended IHC panel

First, the following points should be considered. Tumour types with widely differing phenotypic profiles arise in each organ. Therefore, this article refers to the most common and most clinically relevant in each case. Phenotypic profiles of tumours of the same origin and histological type vary from one case to another, according to their degree of differentiation, and as they progress. This article, therefore, always refers to expression patterns in general, on the understanding that each particular case may display atypical, aberrant, or exceptional phenotypes. As markers can be identified in tumours of different histological types arising in different organs, and practically no absolutely specific markers exist, it is advisable to test for several markers simultaneously to reach a conclusive diagnosis.

Once a first approximation has been obtained using an initial panel (Fig. 1), combined with clinical details (age, gender, past medical and family history, lesion site, biochemistry, imaging, etc.) and the tumour’s histological pattern, thereby confirming that it is a carcinoma and that other tumour types (melanomas, lymphomas, germ line malignancies, etc.) can reasonably be ruled out, specific expression patterns should be studied (Table 1).

**Table 1:** Main phenotypic profiles of the most common tumours

Tumour	Markers
Breast	GATA3, mammaglobin, GCDFP-15, hormone receptors (oestrogens, progesterone, and androgens), CA 15-3, CK34BE12
Salivary gland	Like breast, GFAP
Skin appendages	Like breast, S100
Thyroid	PAX8, TTF1, thyroglobulin
Parathyroid	PTH, GATA3, synaptophysin, chromogranin A
Lung (non-mucinous adenocarcinoma)	TTF1, napsin A, CEA
Lung (mucinous adenocarcinoma)	TTF1, napsin A, CDX2, SATB2-, cadherin 17-, CEA
Cervix (squamous cell)	p40, p63, p16, HPV + , PAX8, 34BE12
Endocervix	CEA, p16, ER/PR-, HPV + , PAX8, vimentin-
Endometrium	PAX8, ER/PR, vimentin, CEA-, WT1-, CA 125
Ovary/fallopian tube (serous)	PAX8, WT1, ER/PR, vimentin, CA 125
Ovary (endometrioid)	PAX8, ER/PR, vimentin, CA 125
Ovary (mucinous)	PAX8, CDX2, WT1-, SATB2-, cadherin 17-, TTF1-
Mesothelium	calretinin, WT1, CK5/6, vimentin, podoplanin (D2-40), CEA-
Colon/rectum	CDX2, SATB2, cadherin 17, villin, AMACR, CEA, CA 19-9
Small intestine	CDX2, cadherin 17, SATB±
Pancreas/bile ducts	CDX2, CK17, cadherin 17, SATB2 ± , CEA, CA 19-9, DPC4-
Stomach	CDX2, CK17-, cadherin 17, SATB±
Liver	arginase-1, Hep Par1, glypican 3
Kidney	PAX8, RCC, carbonic anhydrase IX, glutathione reductase, vimentin, CD10, AMACR, c-KIT
Adrenal	SF1, melan-A, inhibin alpha, synaptophysin, chromogranin A, calretinin
Prostate	NKX3, PSA, AMACR, AR, prostatic acid phosphatase
Squamous cell carcinomas	p40, p63, 34BE12, p16/p53, HPV, MMP-13
Transitional cell carcinomas	p40, p63, GATA3, uroplakin II, S100P, CK34BE12 + , CK8/18
Myoepithelial carcinomas	p63, CK34BE12, myosin, EMA, S100, GFAP, CD10
Neuroendocrine tumours	synaptophysin, chromogranin A, CD56/CD57, PGP9.5, neurofilaments

Underlining indicates markers recommended for their greater specificity and usefulness

## Molecular diagnostics

Thanks to advances in immunohistochemistry, the proportion of CUP cases in which the origin cannot be identified has fallen to 15–20%. This is the patient group most reliant on the use of molecular platforms. Molecular techniques offer information for possible specific therapy. Strategies fall into two categories: platforms for identifying the organ harbouring the primary tumour; and next-generation sequencing to characterise the tumour’s mutation profile.

### Platforms for identifying the organ harbouring the primary tumour

Various molecular platforms exist for evaluating gene expression, microRNA profile or epigenetic pattern in CUP [30–38]. Once a tumour’s molecular profile has been obtained, it is compared against database results from a range of sites and histological types. The molecular profile of the tumour being tested is assessed for similarity to these patterns, and the diagnosis provided suggests one or more sites, each with its estimated probability (similarity score). Table 2 lists the main features of these platforms. The choice of one platform over another depends on several factors, such as availability in different countries. Strategies of more recent introduction tend to be more informative, because they analyse a larger number of possible sites.

**Table 2:** Main molecular diagnostics platforms for cancer of unknown primary

Platform	Method	No. of genes	Sensitivity (%)
Quest-Lab	RT-PCR	92	–
Veridex	RT-PCR	6	76.0
Pathwork	cDNA array	2000	89.0
Cup-print	cDNA array	495	85.0
Rosetta	miRNA array	64	90.0
CancerType^a^	RT-PCR	92	89.0
Epicup^a^	methylation array	–	97.7

^a^Platforms available in Spain

In general, these platforms correlate well with immunohistochemistry, and reduce the number of CUP cases in which the primary cannot be identified. The main difficulties involved in using them are: (1) detecting unusual tumours not always covered by the platforms, e.g., uncommon sites or exceptional histological types; (2) distinguishing between tumours of similar morphology or origin, that may have very similar molecular traits but different oncological management; and (3) to date, these platforms are not funded by the national health system.

The results of these molecular techniques are highly dependent on the quantity, quality, and percentage of tumour cells. Strategies, therefore, need to be established for obtaining optimal material and preserving it after immunohistochemical testing has taken place.

There are a few prospective studies to support the usefulness of these platforms. In a study involving 194 patients, Hainsworth et al. showed that survival was better when the platform predicted tumour types clinically associated with a better response [39]. In general, molecular platforms are regarded as complementary to immunohistochemistry, and useful when a reasonable number of immunohistochemical stains have failed to predict tumour origin, especially in poorly differentiated tumours [34].

### Next-generation sequencing to characterise the tumour’s mutation profile

Sequencing with gene panels enables mutations associated with responses to specific drugs (actionable mutations) to be identified. This strategy has been tested in several studies. Gatalica et al. evaluated 1806 CUP cases by immunohistochemistry (23 markers), sequencing (47 genes) and in situ hybridisation (7 genes) [40]. They found actionable alterations in 96% of cases. In another study, Ross et al. studied formalin-fixed, paraffin-embedded specimens from 200 patients with CUP, 125 of whom had adenocarcinomas [41]. These authors examined 236 genes and the introns of 19 genes often altered in cancer. At least one actionable mutation was identified in 96% of cases. Tothill et al. used panels representing 701 genes and identified actionable mutations in 12 out of 16 cases [42].

The main objection to using massively parallel sequencing in CUP arises from the belief that the potential drug response conferred by a given mutation depends on the type of tumour in which the mutation is found. That is, the context of histological type and tumour site can affect the response to the drug [43]. That assumption is based on studies such as the SHIVA study, which showed that molecularly targeted off-label drug use failed to improve patients’ disease-free survival [44]. Accordingly, sequencing techniques are complementary to platforms for predicting the primary cancer site.

## Targeted clinical work-up and prognosis

The median survival of patients with CUP is approximately 9–10 months, similar to that seen in metastatic lung cancer [45]. A gradual increase has been detected in CUP survival, probably because of improved therapy for malignancies of known origin and better management of metastatic disease, as well as optimisation of diagnostic resources in general [46].

Nevertheless, patients who present with metastasis of a known tumour have better survival than patients whose primary tumour is unknown [47]. Successfully identifying tumour origin improves the patient’s prognosis, probably because it enables better treatment selection and adjustment [48]. That was demonstrated in patients whose CUP molecular profiling results proved consistent with colorectal cancer, and who were treated accordingly (median survival 27 months and 50% response to specific therapy, similar to figures obtained in colon cancer) [49]. Therefore, the factor most likely to improve the prognosis of a patient with CUP is actually not having CUP any more. That is why the algorithm for diagnosing and managing CUP looks for “greatest similarity” to known tumours, in terms of IHC, clinical features, and/or molecular findings.

In the absence of a definitive diagnosis, the clinical work-up should aim to identify tumours that can be treated according to consensus guidelines because of similarity to known primary tumours [2, 25, 50]. Patients with “favourable” CUP have a better prognosis. These cases should be treated with locoregional or systemic therapies, because the survival achieved will be longer and/or similar to their known homologues [51] (Table 3). Unfortunately, these patients only account for 22–23% of cases [52].

**Table 3:** Favourable cancers of unknown primary and specific therapy

Histology	Clinical subset	Further investigation	Therapy
Adenocarcinoma	Female + axillary lymphadenopathies	Breast MRI ER/PR/HER-2	= Breast cancer^a^
	Female + peritoneal carcinomatosis	CA 12.5	= Ovarian cancer^b^
	Male with blastic bone M1 and raised PSA	PSA	= Prostate cancer^c^
	Clinical/pathological features consistent with a primary colorectal tumour	IHC: CK20 +/CK7- and CDX2+	= Colon cancer^d^
	Single M1 lesion	PET	Local therapy ± CT
Squamous cell	Cervical lymph nodes	Endoscopy/PET? Tonsillectomy	= Head and neck cancer^e^
	Inguinal lymph nodes		LND ± RT ± CT
Undifferentiated	Young male, mediastinum and/or retroperitoneum	hCG, AFP	= Extragonadal germ cell cancer^f^
Neuroendocrine	Low or high grade	Octreotide scan	= Neuroendocrine tumour

*AFP* alpha-fetoprotein, *CK* cytokeratin, *CT* chemotherapy, *ER* oestrogen receptors, *hCG* human chorionic gonadotropin, *IHC* immunohistochemistry, *LND* lymph node dissection, *M1* metastasis, *MRI* magnetic resonance imaging, *PET* positron emission tomography, *PR* progesterone receptors, *PSA* prostate-specific antigen, *RT* radiotherapy

^a^Breast cancer: females with adenocarcinoma and axillary lymphadenopathy should be treated as if they had stage II breast cancer

^b^Ovarian cancer: females with peritoneal carcinomatosis should be treated as if they had stage III ovarian cancer, especially in the case of raised CA 12.5, known adenocarcinoma histology, and if gastrointestinal origin has been ruled out

^c^Prostate cancer: males with blastic bone metastases and raised serum prostate-specific antigen should be treated as if they had metastatic prostate cancer

^d^Colorectal cancer: patients whose clinical and pathological features are consistent with a primary colorectal tumour should be treated using the same protocols as for metastatic colorectal cancer, especially in the case of known adenocarcinoma histology and CK20+/CK7- or CDX2+ immunohistochemical staining

^e^Tumours of the head and neck, and anogenital tumours: in patients with squamous cell carcinoma involving the cervical or inguinal lymph nodes only, locoregional approaches based on chemotherapy/radiotherapy strategies are warranted

^f^Extragonadal germ cell tumours: young males with poorly differentiated mediastinal or retroperitoneal tumours should be treated as if they had extragonadal germ cell tumours

The remainder are categorised in the “unfavourable” CUP group, and their prospects depend on various prognostic factors. Their median survival ranges from 3 to 10 months. Clinical, laboratory, and histological prognostic factors have been documented. Poor clinical prognostic factors of particular note are: presenting with multiple metastases (especially at more than 3 sites); and liver, bone, or adrenal metastases [53, 54]. Single sites confer a better prognosis, as do lymph node metastases with no visceral involvement [45]. If only lymph nodes are affected, patients with just the cervical, axillary, and/or inguinal lymph nodes involved have a better prognosis than those with lymph node metastases in pelvic or abdominal locations. Other factors that worsen the prognosis are impaired performance status and previous weight loss [55, 56].

As far as laboratory tests are concerned, common poor prognostic factors are mainly increased lactate dehydrogenase (LDH) and hypoalbuminemia, probably related to weight loss [55, 56]. In terms of haematological findings, leucocytosis and anaemia also constitute independent poor prognostic factors [57]. It is possible that the “inflammation-based” Glasgow Prognostic Score, calculated from the neutrophil/lymphocyte ratio (NLR), may predict with more certainty how a particular case will behave [58].

Among histological factors, adenocarcinomas and undifferentiated tumours have a worse prognosis (3.5% 3-year survival) than squamous cell carcinomas (41.6% 3-year survival) [59].

Petrakis et al. described a prognostic algorithm based on factors that, in their series, independently affected patient survival (Ioannina Score for CUP Outpatient Oncologic Prognostication, I-SCOOP). Those factors were: leucocytosis, CUP subset (visceral disease), and performance status [60]. Using advanced statistical methods, they constructed an algorithm that gave a 5-tier point score. This enabled patients to be categorised into three risk levels: low, intermediate, and high. Other algorithms have been published by various authors, but no consensus has ever been reached, probably because of the heterogeneous nature of the cases included in each series.

## Treating cancer of unknown primary

Treatment strategies for CUP are quite challenging, because these malignancies represent an extremely heterogeneous group of metastatic tumours with, in general, a dismal prognosis. Nevertheless, some subsets of CUP patients, who may harbour chemosensitive and potentially curable tumours, have a favourable risk (about 20%) [25]. Favourable-risk CUP can be identified on the basis of clinical and pathological features, so every effort should be made to identify such cases.

**Fig. 2:**
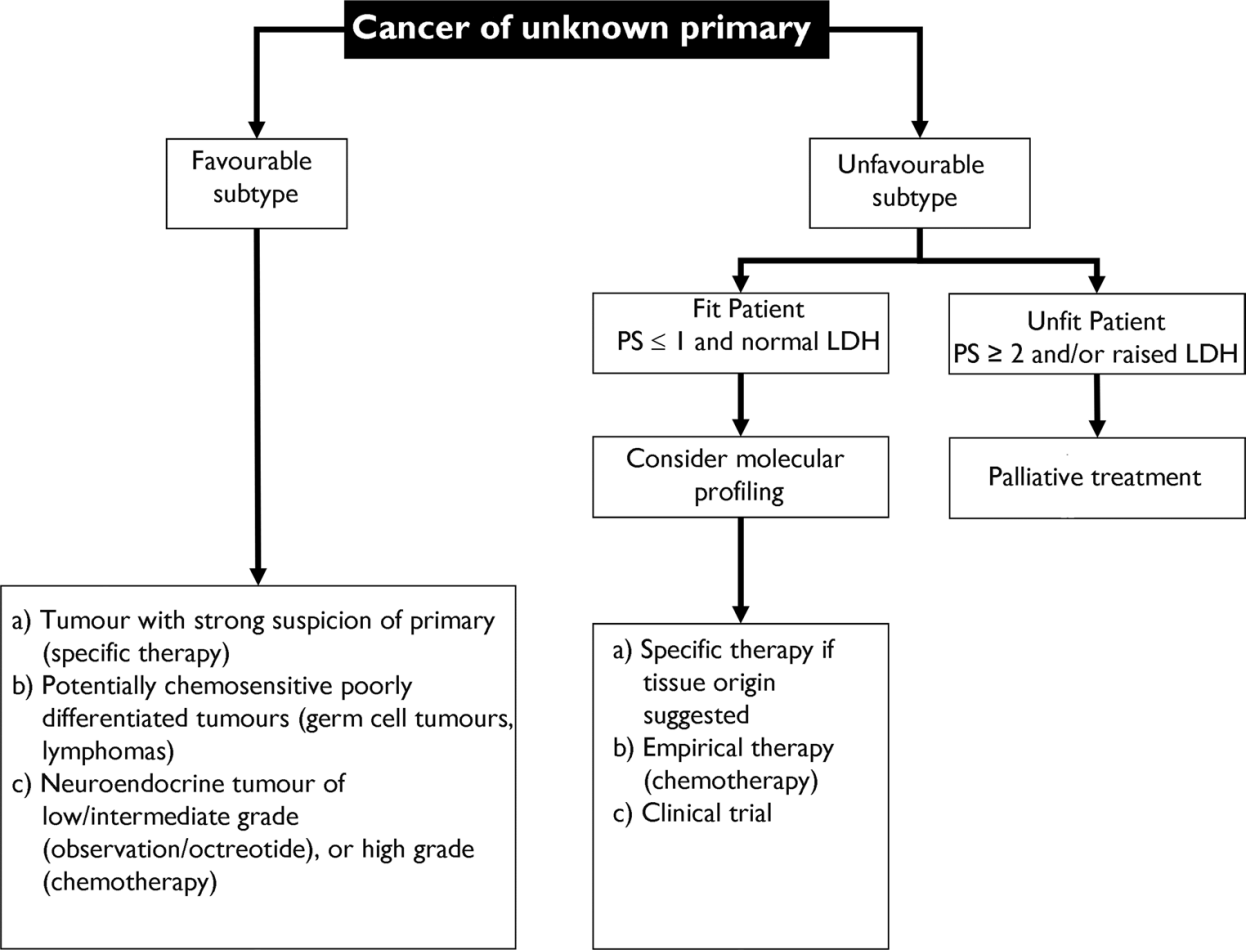
Summary of proposed management of cancer of unknown primary

To establish a rational approach to treatment in this scenario, two main groups can be identified: a) CUP in which the primary site of tumour origin is strongly suspected from the clinical and pathological features; and b) tumours for which no suspicion can be formulated regarding the origin of the primary tumour [2] (Fig. 2).

In the first case, the treatment plan should be based on the same standard strategies established for tumours thought to be primaries [2, 61] (Table 3). As well as the subsets mentioned above, other cases of CUP inviting no suspicions as to primary origin may show a favourable prognosis. That is true of the following patient subsets:

- Poorly differentiated malignancies: up to 60% of these cases are accounted for by lymphomas, which can be suspected on detection of widespread involvement of lymph node territories and organs, such as the liver, and especially the spleen. In these cases, it can be useful to conduct appropriate analysis, including a PET scan and repeated biopsies, if feasible, to guide the use of specific therapies [61].
- Involvement of a single site: locoregional strategies, including surgery, is highly advisable. The use of PET is strongly recommended in this situation, provided not only that limited locoregional CUP is suspected, but also that treatment with curative intent is feasible [3].
- Low/intermediate/high-grade neuroendocrine tumours: these tumours represent a heterogeneous group of malignancies. Well-differentiated neuroendocrine tumours (low and intermediate grades) share similar approaches to diagnosis and treatment. Clinical syndromes may be displayed due to production of hormones or vasoactive substances. Low-grade tumours are often slow-growing, so managing them like well-differentiated neuroendocrine tumours of the gastrointestinal tract is recommended. At advanced stages, the preferred approach is simple observation (asymptomatic patients) or symptomatic treatment with somatostatin analogues (octreotide). Intensive platinum-based chemotherapy seems unhelpful in this context [62]; however, sunitinib or everolimus therapy may be considered when a functioning pancreatic neuroendocrine tumour is suspected [63, 64]. On the other hand, poorly differentiated neuroendocrine tumours are always high-grade tumours (grade 3), with aggressive clinical behaviour and a poor prognosis. These tumours require rapid evaluation and therapy. They typically respond to chemotherapy, so they are treated like disseminated small cell lung cancer. The treatment of choice is chemotherapy cycles of cisplatin and etoposide. This provides overall response rates in the 70–80% range. Other more intensive regimens show a similar response rate but have greater toxicity [62].

Although more consistent evidence is needed, retrospective studies have revealed that favourable-risk CUP patients show clinical behaviour and responses resembling those seen in patients with metastatic tumours of known primary origin [65]. The great majority of CUP patients are categorised in the poor-risk subset. Unfortunately, these patients share a rather dismal prognosis, with median overall survival of 8–12 months from diagnosis [66]. Chemotherapy combinations studied in this context show no evidence of superiority using any regimen [67]. Most of the combinations tested have been based on platinum salts (cisplatin, carboplatin, and oxaliplatin) with other drugs (taxanes, gemcitabine, etoposide, irinotecan, etc.) [68, 69]. However, very modest outcomes have been documented with this approach. It provides symptom relief at best, with no clear overall impact on survival confirmed. Because of its high toxicity and relatively low efficacy in CUP, chemotherapy should always be used judiciously, once other potentially more effective options have been reasonably ruled out (Fig. 2).

In the era of personalised medicine, efforts have also been made to confirm whether there may be a role for targeted therapies in CUP management. The modern gene expression profile assays on the market can identify the origin of CUP tissues in up to 80% of cases [32]. One method for elucidating the origin of a tissue is to select a therapy of proven effectiveness in a class of tumours, and/or to use that information to implement personalised therapies in each patient. This has not yet been tested in clinical trials, but a Phase III prospective trial is being conducted (GEFCAPI04, clinicaltrials.gov NCT01540058).

As well as determining tissue origin based on tumour tissue samples, another promising strategy involves liquid biopsies that detect circulating tumour DNA in patients with CUP. In a recent study of 442 CUP patients, liquid biopsy detected a genomic alteration in 65.6% of cases. The most common mutations were in *TP53*, *KRAS,* and *PIK3CA* [70]. *EGFR* abnormalities, *ERBB2* alterations, and *BRAF* V600E mutations were found in 5.9, 3.6, and 1.6% of cases, respectively. Changes in a mismatch repair (MMR) gene were also detected in 1.6% of cases, making immune checkpoint inhibitor therapy an option. A trial is currently in progress using pembrolizumab in patients with rare tumours, including CUP (clinicaltrials.gov NCT02721732). As well as MMR mutations, other promising biomarkers that may help with immunotherapy, such as tumour mutational burden, are being tested intensively and may play an important role in CUP treatment strategies [71].

## Conclusions

Because of the biological features of CUP, the way in which it spreads, and its aggressive, chemoresistant nature in some cases, it is very important to diagnose it promptly and accurately. Attempts should be made to determine its extent and identify the tumour subtype to which it belongs, to enable use of a specific therapy that has a positive impact on the patient. However, identifying the origin of these tumours is not easy, especially because the same organ can give rise to different tumour types, and tumours with the same origin can vary in phenotypic profile from case to case. Moreover, the literature evidence is sometimes confusing or contradictory, because the diagnostic methods used are not always the same, and results may be interpreted in different ways. It is, therefore, important to have standardised protocols for diagnosis and interpretation of the results.

**Fig. 3:**
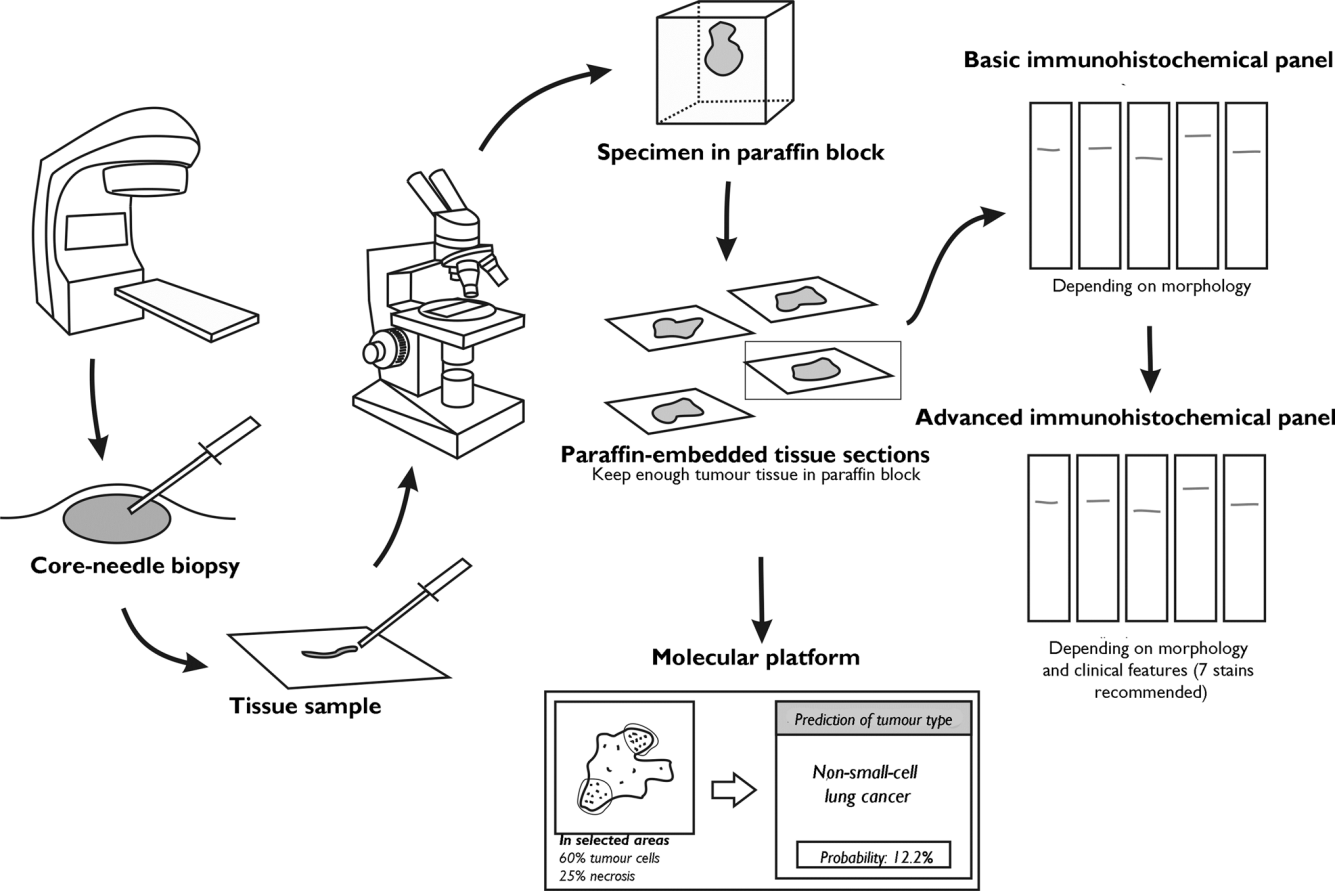
SEAP diagnostic algorithm. Sample material should be obtained by imaging or surgical techniques, preferably in the form of a core-needle, incisional or excisional biopsy. In the pathology department, optimal use of the material should be ensured. This may mean separating fragments into different paraffin blocks to save material for future use, if necessary. Tests should then be done using a basic immunohistochemical panel, according to morphology, and an advanced immunohistochemical panel, based on information obtained from the basic panel, clinical features, and microscopy findings. The recommended number of stains is approximately 7. The aim is to preserve material for possible use of a molecular platform

On the other hand, it is important to obtain a good biopsy, ensuring adequate numbers of tumour cells, and to plan tissue use to obtain as much information as possible. The algorithm proposed by the Spanish Society of Pathology (SEAP) is shown in Fig. 3. The recommendation is to use a basic IHC panel based on the tumour’s clinical and microscopic features, and a specific advanced IHC panel. A limited number of techniques should be performed with both panels, so that a suitable amount of tissue is preserved for using a molecular platform, if thought necessary. Molecular diagnostics and gene expression platforms are considered helpful when used to complement IHC testing, because the results which they provide can be compared against many databases, allowing more accurate diagnosis and specification of tumour origin.
